# Emergence of size-structured dominance hierarchies through size-dependent feedback

**DOI:** 10.1098/rstb.2020.0449

**Published:** 2022-02-28

**Authors:** Ian M. Hamilton, Macie D. Benincasa

**Affiliations:** ^1^ Department of Evolution, Ecology and Organismal Biology, The Ohio State University, 318 W 12th Avenue, Columbus, OH 43210, USA; ^2^ Department of Mathematics, The Ohio State University, 318 W 12th Avenue, Columbus, OH 43210, USA

**Keywords:** body size, strategic growth, dynamic game, growth depensation, feedback

## Abstract

Size-based dominance hierarchies influence fitness, group size and population dynamics and link dominance structure to evolutionary and ecological outcomes. While larger individuals often gain dominance, social status may influence growth and size in return, resulting in feedbacks among status, growth and size. Here, we present two models evaluating how these feedbacks influence the emergence of size structure in a dominance hierarchy. In the first, size influences competition for food and investment in suppressing growth of groupmates. Stable size differences emerged when suppression was greatest for similarly sized individuals and size had little effect on competition for food. The model predicted size divergence when size strongly affected competition for food. In the second model, we used a dynamic game to solve for optimal investment in growth suppression as a function of size structure. Investment in growth suppression was favoured only when dominants and subordinates were similar in size, generating size ratios different than those expected by chance. Variation in the feedbacks among growth, size and status can explain variation in emergent size structure of dominance hierarchies and its consequences for conflict within groups.

This article is part of the theme issue ‘The centennial of the pecking order: current state and future prospects for the study of dominance hierarchies’.

## 1. Introduction

In many animals, dominance is strongly correlated with relative body size, with larger individuals dominant to smaller ones (e.g. [1–3]). This size structure can influence conflict within groups (e.g. [4]), the maximum sizes of groups [5,6] and the population dynamics of organisms that live in size-structured hierarchies [5,7,8]. Dominance results from repeated agonistic interactions among individuals [9] and, as size can determine resource holding potential [10,11], large size may allow individuals to gain and maintain status through such interactions. A relationship between large size and better fighting ability has been found in a wide range of taxa, including but not limited to spiders (e.g. [12]), insects (e.g. [13]), fishes (e.g. [14,15]), amphibians (e.g. [16]), birds (e.g. [17]) and mammals (e.g. [18,19]), including in species that habitually form long-term, dominance-structured groups (e.g. [20]). However, while relative size can influence status, status can influence growth and size in return (e.g. [2,21,22]), particularly in organisms with indeterminate growth or in growing juveniles. Therefore, understanding the causes and consequences of size structure in dominance hierarchies requires understanding feedbacks among status, size and growth.

Feedback between growth and size can arise in dominance-structured societies if higher status individuals have better access to food resources while those with lower status are excluded [23–27]. Given that size, in turn, affects competitive ability, this can result in a positive feedback between size and ability to monopolize food, leading to continued size divergence between larger, more dominant individuals that have superior access to food resources and smaller subordinates excluded from food resources (growth depensation; [7,8,28–31]). These emergent competitive asymmetries can result in strong selection on emergence timing in juvenile fishes [32] and can have population-level impacts as smaller, subordinate individuals are forced to emigrate or search for food in potentially less desirable feeding locations, increasing their exposure to predation risk [7,8,33].

An additional feedback between growth and size can arise in dominance-structured societies because of size-related conflict over growth. Dominants may gain from suppressing the growth of similarly sized subordinates (or causing subordinates to restrain their growth) by reducing the risk of being displaced from the dominant position or the costs of reproductive competition when size differences are small. Investment in suppressing the growth of other group members may be costly, for example by increasing stress hormone levels [34] or risks of group dissolution [22]. However, such growth effects do not necessarily require that dominants exclude subordinates from resources. Subordinates can gain from restraining their own growth by reducing the costs and risks associated with size-related conflict. Because large size is a strong determinant of fighting ability [20], reproductive success [2,35,36] and social status [2,22,37–39] in many systems, smaller individuals must tradeoff the benefits of rapid growth with the risks of triggering costly conflict and eviction by larger, more powerful individuals. The net benefits of suppressing or restraining growth may be highest when sizes are similar, giving rise to close-competitor strategies (e.g. Dehnen *et al*. [40]) and resulting in negative feedback between the strength of suppression (or restraint) and size differences in the group.

Several group-living teleost fishes exhibit a pattern of growth consistent with such a negative feedback, which is sometimes referred to as ‘strategic growth’ (e.g. [4,21]). Unlike the continual divergence of relative growth rates characteristic of growth depensation, growth rate in these fishes is socially regulated such that smaller, subordinate individuals grow rapidly when the size difference with a larger, more dominant groupmate is large, and slowly when this size difference is small (e.g. *Neolamprologus pulcher* [4,21]*; Amphiprion percula* [2,41]*; Paragobiodon xanthosomas* [22]*, Centropyge bicolor* [42]). This growth pattern has been suggested to result in the emergence of stable, unequal size structure in dominance hierarchies, such that dominant individuals are larger than subordinates, size differences between adjacently ranked individuals are consistent over time, and size differences are different from those expected by chance [5]. Dominant and subordinate individuals may benefit from this stable, unequal size structure by reducing the risks and costs associated with size-related conflict [2]. In several species exhibiting strategic growth, small size differences are associated with increased aggression received by subordinates, a higher probability of eviction from the group and fewer affiliative behaviours [5,22,43]. In at least one system, strategic growth appears to result from subordinate self-restraint; in the goby *P. xanthosomas*, subordinates refrain from eating when size differences with dominants are small and probability of eviction is high [44].

Growth patterns and emergent size structure in dominance hierarchies can differ within and among societies. For example, resource abundance influences whether growth depensation is observed in zebrafish (*Danio rerio* [45]). In the cooperatively breeding cichlid, *N. pulcher*, strategic growth occurs among males but is weak or non-existent among females [4,46]. For males, but not females, size differences between adjacently ranked individuals are different than expected by chance [4]. Further, the positive and negative feedback mechanisms outlined above are not mutually exclusive, and so the relative strengths of these might give rise to qualitatively different patterns of size structuring among societies. As variation in size structure can affect individual fitness and have group and population-level consequences [4–8], understanding how and when such variation arises is important for understanding the evolutionary and ecological consequences of dominance structure.

To evaluate how and when feedbacks among size, growth and status results in the emergence of size structure in dominance hierarchies, we developed two dynamic models of growth in dominance-structured systems with indeterminate growth. The goals of these models were to: (i) explain variation in size structure by identifying the conditions under which size-based competition for food and size-based growth suppression result in the emergence of patterns such as growth depensation or stable but unequal size structure, (ii) find optimal patterns of investment in growth suppression and their consequences for size structuring in dominance-structured groups, and (iii) assess the consequences of emergent size structure on individual- and group-level performance as measured by individual fitness, costly competition over growth and eviction. These models are based on the growth patterns of social teleost fishes such as juvenile salmonids (e.g. [8]) or cooperative breeding cichlids (e.g. [21]), but the model is intended to be general and applicable to other organisms with indeterminate growth or to growing juveniles. In the first model (Model 1), group members compete for food and invest in suppressing the growth of groupmates, both of which influence growth rate. In turn, relative size influences success at size-based competition for food and ability to suppress partner growth. In the first model, patterns of growth suppression are specified in the model and unable to evolve. However, the decision to suppress the growth of a groupmate has fitness consequences as discussed earlier. Because optimal strategies could change given absolute and relative size and temporal proximity to reproductive events, as well as depend on the expected strategies of partners, we used a discrete stochastic dynamic game (Model 2) to predict the size ratios at which dominant and subordinate individuals attempt to suppress partner growth and the consequences of these policies on size ratios at the evolutionary stable strategy (ESS). In addition, we use Model 2 to assess the effects of evolutionarily stable suppression decisions on the risk of group dissolution.

## 2. The models

### (a) Model 1: a model of growth incorporating size-based suppression and competition for food

All model parameters are listed in the electronic supplementary material, table A.1.

#### (i) Model description

In this model, a group consists of two individuals: a subordinate (s) and a dominant (d). These have masses *m*_s_ and *m*_d_, respectively. The size ratio of the pair, *r* = *m*_s_*/m*_d_. Without loss of generality, we assume that *m*_s_ ≤ *m*_d_ in this model. Model 1 is deterministic and so it is not possible for the subordinate to exceed the dominant in size given the initial conditions *m*_s_ ≤ *m*_d_.

In the absence of conflict, growth of individual i = {s, d} follows a von Bertalanffy growth equation:

2.1 $$\frac{dm_{i}}{dt}=Am_{i}^{b}-Dm_{i}^{\gamma }.$$

In equation (2.1), *A* is an anabolic constant, which is assumed to be proportional to consumption rate, and *D* is a catabolic constant. For simplicity, we set *γ* = 1. We assume asymptotic growth, with *b* < 1.

Growth rate is influenced by the characteristics of the pair because of (i) suppression or self-restraint of growth resulting from size-based conflict that is most intense when individuals are similar in size, and (ii) size-based competition for food, in which larger individuals are superior competitors. Size-based conflict between individuals i and j influences j's growth by reducing the anabolic constant (*A*) by some proportion *U*_ji_. We do not specify the mechanism underlying growth suppression; growth regulation could result through self-restraint [44], shifts in energy allocation [47] or by individuals directly preventing growth of their partners in some way (e.g. excluding them from resources when similar in size [26]). We model the effect of conflict on growth, *U*_ji_ as a function of the state of conflict between dominant and subordinate over growth, the investments of each individual in conflict over growth and the consequences of each individual's investment in conflict on suppressing the growth of itself and its partner.

The state of conflict over growth, 0 ≤ *u* ≤ 1, represents the maximum opportunity for individuals to suppress the growth of their partners. For the purposes of the model, we do not specify the mechanisms that influence *u*, but these could include the intrinsic conflicts of interest within the pair and the pair's opportunities to interact.

Although there may be an opportunity for conflict, this does not necessarily result in suppression. The investment of individual i in suppressing the growth of its partner is *f*_i_:

2.2 $$f_{i}=f_{j}=\frac{h}{\cosh{\left(x(r-1)\right)}^{2}}.$$

Equation (2.2) describes a bell-shaped function with a maximum, *h*, at *r* = 1. We assume that *h* ≤ 1. The parameter, *x*, influences the effects of relative size on this investment and can be thought of as a measure of the importance of strategic growth. If *x* is large, then investment is high only when partners are similar in size. When *x* is 0, relative size has no effect on investment in suppressing growth of partners. We assume that such investment can be costly to the growth of i and increases *U*_ij_ (the decrease in i's growth as a result of size-based suppression) by some amount *qf*_i_.

Individual i's investment in suppressing growth, *f*_i_, increases the suppression of its partner's growth, *U*_ji_ by some amount *Cf*_i_, independent of j's behaviour. In addition, if *f*_i_ > 0 and *f*_j_ > 0, there is a costly tug-of-war [48] over growth that reduces the growth rate of both partners, although not necessarily by equal amounts. The outcome of this tug-of-war is described by *g*_s_, which is a function of relative size. We assume that *g*_s_ + *g*_d_ = 1 and so, *g*_d_ = 1 − *g*_s_. The value of *g*_s_ follows a sigmoid function that has a value of 0.5 when subordinates and dominants are equal in size and approaches 1 when dominants are much larger than subordinates:

2.3 $$g_{s}=\frac{1}{2}(1+\tanh(y(1-r))).$$

Equation (2.3) has an inflection point at *r* = 1, at which growth suppression resulting from the tug-of-war is the same for both partners. The value of *y* influences the slope of *g*_s_ at the inflection point. If *y* is large, then *g*_s_ rapidly approaches 1 as subordinate size, relative to dominant size, decreases. The parameter *z* scales suppression resulting from the tug-of-war to *C* such that the total effect of a tug-of-war on i's growth is *g*_i_*zC*.

From above, size-based suppression of individual i's growth, *U*_ij_, is:

2.4 $$U_{\mathrm{ij}}\;=\;u\{qf_{i}+Cf_{j}(1+zf_{i}g_{i})\}.$$

Relative size can also influence growth through size-based competition for food, in which larger individuals are superior competitors. To incorporate size-based competition for food, success at competition modifies the anabolic component of growth rate through the following function:

2.5 $$\theta_{i}=2\frac{m_{i}^{k}}{m_{s}^{k}+m_{d}^{k}}.$$

In equation (2.5), *k* is a measure of the effect of size on competitive ability (*k* ≥ 0). If *k* = 0, then size has no effect on competition for food and the value of *θ*_i_ for each individual is 1. If *k* > 0, then smaller individuals are poorer competitors and grow more slowly, all else being equal.

Incorporating size-based suppression and competition for food into the growth equation, the growth rates of the subordinate and dominant are:

2.6*a* $$\frac{dm_{s}}{dt}=\theta_{s}(A-AU_{\mathrm{sd}})\;m_{s}^{b}-Dm_{s}$$

and

2.6*b* $$\frac{dm_{d}}{dt}=\theta_{d}(A-AU_{\mathrm{ds}})\;m_{d}^{b}-Dm_{d},$$

and the change in the size ratio over time is:

2.6*c* $$\frac{dr}{dt}=\frac{(dm_{s}/dt)m_{d}-(dm_{d}/dt)m_{s}}{m_{d}^{2}}.$$

We use equations (2.6*a*–*c*) to find equilibrium size ratios at which the size ratio does not change (i.e. dr/dt=0) and evaluate the dynamic stability of these equilibria. At the equilibrium size ratio, r*, both individuals continue to grow but their sizes relative to one another do not change over time.

#### (ii) Results of Model 1

**Figure 1.**
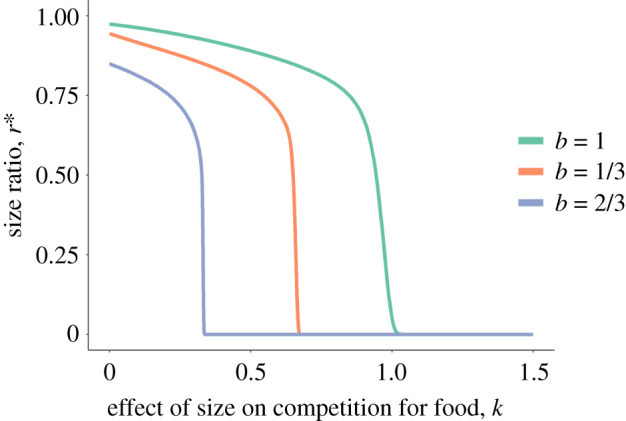
Bifurcation plots of equilibrium size ratios, r* plotted against the effect of relative size on competition for food, *k* in Model 1. If *k* > 0, larger individuals are superior competitors. The lines represent the stable equilibrium point to the model for a given value of *k*; there is only ever one stable equilibrium point for any value of *k.* As the value of *k* increases, the stable equilibrium size ratio shifts from greater than 0 to 0 at *k* = 1 − *b* (panel *a*). Results are plotted for three different values of *b*, a measure of nonlinearity of the effect of size on growth rate. Other model parameters are: *A* = 0.05, *D* = 0.00323, *q* = 0.05, *z* = 0.5, *C* = 0.5, *h* = 1, *u* = 1, *x* = 1, *y* = 3. (Online version in colour.)

In Model 1, there always exist at least two equilibrium size ratios (r*) at which d*r*/d*t* = 0 on the domain *0* ≤ *r* ≤ 1. There is always an equilibrium point at $r_{0}^{*}=0$. While $r_{0}^{*}$ is biologically unrealistic as it would require the mass of the smaller individual to be 0, a system in which the only stable fixed point is at $r_{0}^{*}$ represents a situation in which size differences continually increase over time. In numerical solutions, this fixed point was always stable $\left((d^{2}r/dt^{2})<0\right)$ when the exponent that relates ability to compete for food with size, *k* > 1 − *b* (figure 1)*.* Further, if *k* > 1 − *b*, the only stable equilibrium point in the system was at $r_{0}^{*}=0$ (figure 1). The point $r_{0}^{*}=0$ was also the only stable equilibrium point in the system when *x* *=* 0 and an equilibrium size ratio of 1 was not stable (see below). Varying other model parameters had no effect on the stability of $r_{0}^{*}$ (I.M. Hamilton, M.D. Benincasa 2021, unpublished modelling results).

There also always exists an equilibrium point at $r_{e}^{*}=1$; i.e. when the sizes of the two individuals are equal. Finally, because d*r*/d*t* is continuous over the domain 0 ≤ *r* ≤ 1, then if both $r_{0}^{*}$ and $r_{e}^{*}$ are unstable fixed points, there must also exist at least one stable fixed point $r_{u}^{*}$ with a value between 0 and 1. This point, $r_{u}^{*}$, never existed in numerical solutions when *x* = 0. At $r_{u}^{*}$, the equilibrium sizes of the two individuals are unequal. In numerical solutions to the basic model, whenever $r_{u}^{*}$ existed, then it was the only fixed point between 0 and 1, it was always stable, and $r_{e}^{*}$ was unstable. Conversely, if $r_{u}^{*}$ did not exist, then $r_{e}^{*}$ was stable when $r_{0}^{*}$ was unstable (*k* < 1 – *b;* figure 1). The fixed point at $r_{e}^{*}$ is unstable (and therefore, $r_{u}^{*}$ exists and is stable) when *x* > 0, $r_{0}^{*}$ is unstable, and:

2.7 $$y>(1-b-k)\left(\frac{1}{uzh^{2}C}-\frac{1}{zh}-\frac{q}{zhC}-\frac{1}{2}\right).$$

**Figure 2.**
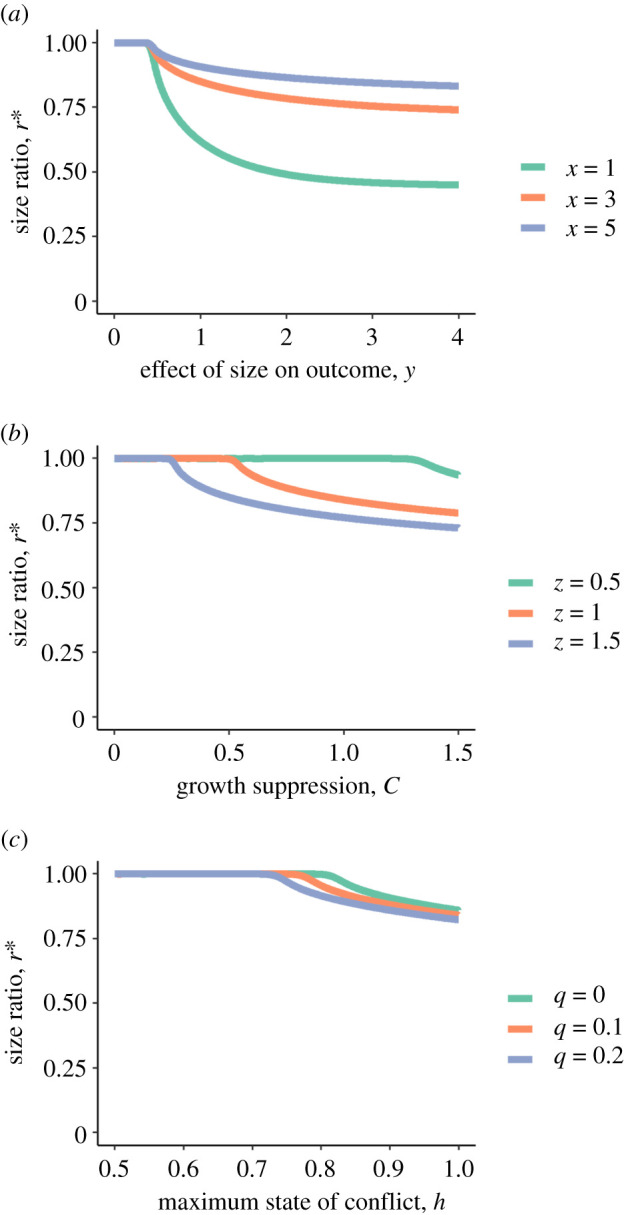
Bifurcation plots of equilibrium size ratios, r* plotted against various bifurcation parameters for Model 1. The lines represent stable equilibrium points to the model for a given value of the bifurcation parameters; there is only ever one stable equilibrium point for any combination of parameters*.* As the value of *y, C* or *h* increase, the stable equilibrium size ratio shifts from 1 to less than 1. For each panel, results are plotted for three values of a second bifurcation parameter. For example, in (*b*), the value of *C* at which the stable size ratio shifts from 1 to less than 1 depends on the value of *z.* (*a*) Size ratios are plotted against the effect of relative size on the outcome of a tug-of-war over growth, *y*, for three values of *x*, the effect of relative size on the probability of attempting to suppress partner growth. (*b*) Size ratios are plotted against ability to suppress growth, *C*, for three values of *z*, which influences growth suppression resulting from the tug-of-war. (*c*) Size ratios are plotted against the maximum investment in growth suppression, *h*, for three values of the growth cost of investing in suppression, *q*. Unless otherwise stated, other model parameters are: *A* = 0.05, *D* = 0.00323, *q* = 0.05, *z* = 0.5, *C* = 0.5, *h* = 1, *u* = 1, *x* = 1, *y* = 3. (Online version in colour.)

In inequality (2.7), *z, h, C* and *u* all appear in the denominator, and so must be greater than 0. All of these parameters influence the ability and opportunity to suppress partner growth. In figure 2, we show bifurcation plots of the effects of varying several model parameters on the existence and stability of $r_{e}^{*}$ and $r_{u}^{*}$. Increasing the effect of relative size on the outcome of the tug-of-war over growth (*y,* figure 2*a*), ability to suppress growth (*C* and *z*, figure 2*b*), maximum investment in suppression (*h,* figure 2*c*) and the growth cost of investment (*q,* figure 2*c*) can shift the stable equilibrium from equal sizes to unequal sizes. When $r_{u}^{*}$ exists, further increases in these parameters lead to smaller size ratios at equilibrium (i.e. the relative difference in size becomes larger). The effect of relative size on investment in suppression (*x*) does not affect whether $r_{u}^{*}$ exists when *x* > 0 (figure 2*a*). However, as *x* increases, equilibrium size ratios become closer to 1.

In summary, the emergence of unequal size ratios at equilibrium in this model requires that three conditions must be met. First, the value of *k*, which relates size to ability to compete for food, must be sufficiently small that size ratios do not diverge over time for all size ratios. Second, investment in suppression must be size dependent (*x* > 0). Third, the values of the parameters that influence the opportunity and ability to suppress partner growth (*y, h, u, q*, z and *C*) must be sufficiently large that $r_{u}^{*}$ exists.

### (b) Model 2: an evolutionary model of growth suppression

#### (i) Model description

The model includes two players (1 and 2) of sizes *m*_1_(*t*) and *m*_2_(*t*) (hereafter, *m*_1_ and *m*_2_) that are in competition over social status, and as a consequence, opportunities for reproduction, at the end of the game. Size is a discretized variable with range {*m*_min_, *m*_max_}. By definition, player 1 has status i = s if *m*_1_ < *m*_2_ and i = d if *m*_1_ > *m*_2_. Player 2 has status i = d if *m*_1_ < *m*_2_ and i = s if *m*_1_ > *m*_2_. If sizes are equal, each individual retains its status from the previous time step. In the following presentation of the model, it is often more intuitive to refer to the size of the subordinate (*m*_s_) and dominant (*m*_d_) at a particular time than of the sizes of individuals 1 and 2. At any given time, *m*_s_ = *m*_1_ and *m*_d_ = *m*_2_ if player 1 has status s at that time, and *m*_s_ = *m*_2_ and *m*_d_ = *m*_1_ otherwise. Changes in size and behaviour are modelled in discrete time, with a maximum time *t* = *T*, at which reproduction occurs.

We reformulate Model 1 so that events are probabilistic, but the basic structure of Model 1 is retained. To focus on growth suppression, we assume for Model 2 that *k* = 0, so that there is no size-based competition for food and *θ* = 1 for both individuals. In a given time step, the following events can occur: an opportunity to suppress arises with probability *u*, the subordinate is willing to suppress its partner with probability, *f*_s_(*m*_s_, *m*_d_, *t*) = *f*_s_, the dominant is willing to suppress its partner with probability, *f*_d_(*m*_s_, *m*_d_, *t*) = *f*_d_, the subordinate wins the tug-of-war if both attempt suppression with probability *g*_s_(*m*_s_, *m*_d_, *t*) = *g*_s_, and the subordinate is evicted after losing the tug-of-war with probability *v*. In this model, *g*_s_ is the same as in equation (2.3), while *f*_s_ and *f*_d_ are the objectives of the model. Thus, there are six possible states in the model: no suppression (*ϕ* = 1), one-sided suppression in which the subordinate invests in suppression and the dominant does not (*ϕ* = 2), one-sided suppression with the dominant, but not the subordinate, investing in suppression (*ϕ* = 3), a tug-of-war with the subordinate winning (*ϕ* = 4), a tug-of-war with the dominant winning and the subordinate remaining in the group (*ϕ* = 5), and a tug-of-war with the dominant winning and the subordinate being evicted (*ϕ* = 6). These occur with probabilities

2.8 $$p(\phi )=\begin{array}{c} \left(1-u)+u(1-f_{s})(1-f_{d}\right)\\ uf_{s}(1-f_{d})\\ \begin{array}{c} uf_{d}(1-f_{s})\\ uf_{s}f_{d}g_{s}\\ \begin{array}{c} uf_{s}f_{d}(1-g_{s})(1-v)\\ uf_{s}f_{d}(1-g_{s})v \end{array} \end{array} \end{array}\;for\;\phi =\{\begin{array}{c} 1_{}\\ 2_{}\\ \begin{array}{c} \begin{array}{c} 3_{}\\ 4_{}\\ 5_{} \end{array}\\ 6_{} \end{array} \end{array}.$$

Changes in size of the dominant and subordinate are discretized from equations (2.1)–(2.6) in Model 1 using Euler's forward method as follows:

2.9 $$\Delta m_{i,\phi }=\varepsilon G_{i,\phi }(m_{s},\;m_{d}).$$

In equation (2.9), *ε* is the length of the discrete time step. *G* is a continuous time growth function that depends on the events occurring in a given time step:

2.10*a* $$\begin{array}{r} G_{s,\phi }(m_{s},\;m_{d})=\begin{array}{c} Am_{s}^{b}-Dm_{s}\\ A(1-q)m_{s}^{b}-Dm_{s}\\ \begin{array}{c} A(1-C)m_{s}^{b}-Dm_{s}\\ A(1-C-q)m_{s}^{b}-Dm_{s}\\ \begin{array}{c} A(1-C-zC-q)m_{s}^{b}-Dm_{s}\\ A(1-C-zC-q)m_{s}^{b}-Dm_{s} \end{array} \end{array} \end{array}\;for\;\;\phi =\{\begin{array}{c} 1_{}^{}\\ 2_{}^{}\\ \begin{array}{c} \begin{array}{c} 3_{}^{}\\ 4_{}^{}\\ 5_{}^{} \end{array}\\ 6_{}^{} \end{array} \end{array} \end{array}$$

and

2.10*b* $$\begin{array}{r} G_{d,\phi }(m_{s},\;m_{d})=\begin{array}{c} Am_{d}^{b}-Dm_{d}\\ A(1-C)m_{d}^{b}-Dm_{d}\\ \begin{array}{c} A(1-q)m_{d}^{b}-Dm_{d}\\ A(1-C-zC-q)m_{d}^{b}-Dm_{d}\\ \begin{array}{c} A(1-C-q)m_{d}^{b}-Dm_{d}\\ A(1-C-q)m_{d}^{b}-Dm_{d} \end{array} \end{array} \end{array}\;for\;\;\phi =\{\begin{array}{c} 1_{}^{}\\ 2_{}^{}\\ \begin{array}{c} \begin{array}{c} 3_{}^{}\\ 4_{}^{}\\ 5_{}^{} \end{array}\\ 6_{}^{} \end{array} \end{array}. \end{array}$$

Parameters *C*, *z* and *q* are the same as their equivalents in Model 1. There is no additional growth cost to being evicted (*ϕ* = 6), but subordinates that are evicted also experience suppression from their partner before eviction (equation (2.10*b*)). If the resulting size at time *t* + 1 is a non-integer, we use linear interpolation to generate a probability distribution of sizes at time *t* + 1.

Fitness for the subordinate (the smaller individual) is denoted *w* and fitness for the dominant (the larger individual) is denoted *W*. At *t* = *T*, individuals gain fitness proportional to their size:

2.11*a* $$w(m_{1},\;m_{2},\;T)=\;\alpha_{s}m_{s}$$

and

2.11*b* $$W(m_{1},\;m_{2},\;T)=\;m_{d}.$$

The rate of fitness increase with size is assumed to be lower for subordinates than dominants (*α*_s_ < 1).

At *t* < *T*, the fitness pay-offs for a given strategy of *f*_i_ ($w_{\;f_{s}}$ and $W_{\;f_{d}}$) depend on current size and the expected size of the dominant and subordinate in the next time step. If the subordinate in one time step is still the smallest individual in the next (or sizes are equal), it remains subordinate; otherwise, the erstwhile subordinate becomes dominant in the next time step. Therefore, the probability that there is a change in status is *λ_ϕ_* = 1 if *m*_s_ + Δ*m*_s,*ϕ*_ > *m*_d_ + Δ*m*_d,*ϕ*_ and *λ_ϕ_* = 0 otherwise. If an individual is evicted or evicts another, it becomes a dominant in a new group (with a subordinate of size *m*_n_) with probability *l*_i_. Fitness at any time *t* < *T* is:

2.12*a* $$\begin{array}{rl} w_{\;f_{s}}(m_{s},\;m_{d},\;t) & =p(6)l_{s}W(m_{n},m_{s}+\Delta m_{s,6},t+1)\\ & \;+\overset{5}{\underset{\phi =1}{\sum }}p(\phi )[\left(1-\lambda_{\phi })w(m_{s}+\Delta m_{s,\phi },m_{d}+\Delta m_{d,\phi },t+1\right)\\ & \;\;+\;\lambda_{\phi }W(m_{d}+\Delta m_{d,\phi },m_{s}+\Delta m_{s,\phi },t+1)]\; \end{array}$$

and

2.12*b* $$\begin{array}{rl} W_{\;f_{d}}(m_{s},\;m_{d},\;t) & =p(6)l_{d}W(m_{n},m_{d}+\Delta m_{d,6},t+1)\\ & \;+\overset{5}{\underset{\phi =1}{\sum }}p(\phi )[\lambda_{\phi }w(m_{d}+\Delta m_{d,\phi },m_{s}+\Delta m_{s,\phi },t+1)\\ & \;\;+(1-\lambda_{\phi })W(m_{s}+\Delta m_{s,\phi },m_{d}+\Delta m_{d,\phi },t+1)]. \end{array}$$

All else being equal, individuals gain from large size and high social status. Equation (2.12) assumes implicitly that grouping is beneficial (because fitness is 0 if a newly solitary individual fails to found a new group). Equation (2.12) also implies that individuals do not queue for social status. Individuals cannot wait for a dominant to die and then inherit dominant status; they can only gain status by leaving the group or by outgrowing the other individual. Finally, all reproductive success is gained at time *T*, which is fixed for all individuals; an individual that newly gains dominant status after eviction does not start at *t* = 1.

Fitness for individuals 1 and 2 playing strategy *f*_i_ ($\omega_{1,f_{i}}$ and $\omega_{2,f_{i}}$) are:

2.13*a* $$\omega_{1,f_{i}}(m_{1},\;m_{2,\;}t)=\{\begin{array}{c} w_{\;f_{i}}(m_{1},\;m_{2},\;t)\\ W_{\;f_{i}}(m_{2},\;m_{1},\;t) \end{array}\;\;\begin{array}{c} \mathrm{if}\;i=s\\ \mathrm{otherwise} \end{array}$$

and

2.13*b* $$\omega_{2,f_{i}}(m_{1},\;m_{2,}\;t)=\{\begin{array}{c} w_{\;f_{i}}(m_{2},\;m_{1},\;t)\\ W_{\;f_{i}}(m_{1},\;m_{2},\;t) \end{array}\;\;\begin{array}{c} \mathrm{if}\;i=s\\ \mathrm{otherwise} \end{array}.$$

The model was solved using iterated backward induction [49] as described in the electronic supplementary material, B. We used the results of backward induction to forward simulate 10 000 pairs of dominants and subordinates (see the electronic supplementary material, B). We measured the final size ratio and whether eviction occurred for each pair.

#### (ii) Results of Model 2

**Figure 3.**
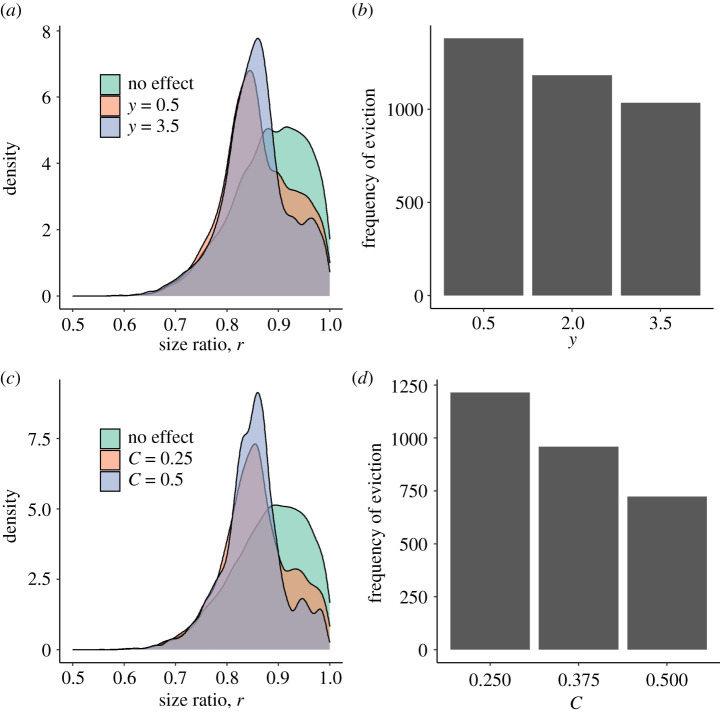
Final size ratios (*a*,*c*) and frequency of evictions (*b*,*d*) in *n* = 10 000 forward simulations of Model 2, in which decisions about growth suppression can evolve. ‘No effect’ refers to simulations in which conflict had no effect on growth and there was no probability of eviction. Top (*a*,*b*): the effect of relative size on the outcome of a tug-of-war over growth, *y*. Bottom (*c*,*d*): the effect of suppression on partner growth, *C*. Unless otherwise specified, *A* = 0.05, *C* = 0.25, *D* = 0.00323, *α*_s_ = 0.5, *b* = 2/3, *z* = 0.5, *u* = 1, *y* = 2, *h* = 0.8, *q* = 0.02, *T* = 25, *ε* = 1, *m*_max_ = 100, *v* = 0.05, *l*_d_ = 0.9, *l*_s_ = 0.5, *m*_n_/*m*_d_ = 0.5. (Online version in colour.)

The model converges to an ESS over a wide range of parameters (see parameter values on figure 3; electronic supplementary material, C). Model convergence was not achieved in all cases, for example when values of *y* or *q* were 0 and when the probability and costs of eviction were high (e.g. *v* was high or *l*_d_ was low). At the ESS in Model 2, both subordinates and dominants invest in suppression only when size differences are small. As shown in the electronic supplementary material, figures C.1–C.3, suppression was found only when the difference between *m*_s_ and *m*_d_ was small. For most parameters, subordinates invested in suppression over a smaller range of relative sizes than dominants (electronic supplementary material, figures C.1–C.3).

The distribution of size ratios in our forward simulations differed between simulations in which fighting influenced growth and those in which it did not (figure 3; electronic supplementary material, figures C.4 and C.5), with the former exhibiting a narrower distribution of size ratios and fewer size ratios close to 1. Variation in most model parameters had weak effects on simulated size ratios (figure 3; electronic supplementary material, figure C.4). Increasing the risks of losing (e.g. *y*; figure 3*a*) or the costs of suppression (e.g. *C:* figure 3*c*; *z* and *α*_s_; electronic supplementary material, figure C.4) resulted in narrower variation in size ratios, with fewer simulated pairs having size ratios close to 1. Increasing *q*, the cost of investing in suppression on the suppressor's growth also resulted in fewer pairs with size ratios close to 1 but a mode of the size distribution closer to 1. Increases in all of the aforementioned variables resulted in less frequent eviction from the group. By contrast, increasing *u*, the probability of an opportunity to suppress partner growth, had little effect on size-ratio distributions and resulted in increased frequency of eviction (electronic supplementary material, figure C.4).

Similarly, varying parameters related to the likelihood and cost of eviction had mostly weak effects on simulated size ratio distributions (electronic supplementary material, figure C.5). Increasing the likelihood of eviction upon loss, *v*, or the likelihood that subordinates gain dominant position after eviction, *l*_s_, resulted in fewer pairs with size ratios close to 1. Varying the size of subordinates newly attracted to a group after eviction (*m*_n_/*m*_d_) had little effect on simulated size ratios. However, the probability that dominants would attract a new subordinate after eviction, *l*_d_, had a strong influence on the shape of the size-ratio distribution. When this value was sufficiently low, the size-ratio distribution was bimodal; there was a peak at size ratios close to 1 and another peak at size ratios much smaller than 1 (electronic supplementary material, figure C.5). Increasing the likelihood of eviction upon loss, *v*, resulted in fewer evictions (when *v* *>* 0; electronic supplementary material, figure C.5). Increases in *l*_s_ or *l*_d_ resulted in increased frequency of eviction, while eviction was less frequent as the ratio *m*_n_/*m*_d_ increased (electronic supplementary material, figure C.5).

### (c) Discussion

Three patterns of size structure emerged in dominance-structured groups in Model 1: a stable size ratio of 1 ($r_{e}^{*}$ is stable), a stable size ratio of 0 ($r_{0}^{*}$ is stable) or a stable size ratio between 0 and 1 (neither $r_{0}^{*}$ nor $r_{e}^{*}$ is stable; $r_{u}^{*}$ is stable). For all parameters explored, one and only one of these points is stable. In real systems, it is unlikely that all individuals will be the same size even though $r_{e}^{*}$ is stable, owing to individual variation in size and growth. However, if this is the case, differences in size should reflect random variation rather than a systematic size difference between dominants and subordinates [5]. While $r_{0}^{*}$ is biologically unrealistic as it would require the mass of the smaller individual to be 0, this point represents a situation in which size differences continually increase over time (i.e. *r* approaches 0 over time), consistent with growth depensation. Finally, at $r_{u}^{*}$, size differences should be different from those expected by chance [5] and the system should return to $r_{u}^{*}$ if size ratios are perturbed (e.g. [22]).

Growth depensation was found in the model when larger individuals had a strong advantage in competition for food (*k* > 1 − *b*) (figure 1). Such size divergence occurs because of positive feedback between size and competitive ability, in which large individuals are better able to monopolize food and thereby grow faster. This growth pattern has been observed in a diversity of systems with and without dominance structure, including groups of fishes competing for food (e.g. [28,29,31]), bird chicks in competition for parental provisioning (e.g. [50]) and plants in competition for sunlight (e.g. [51]). By contrast, if relative size had a weak effect on ability to compete for food, then individuals either all grew to the same size or to a stable but unequal size ratio. This prediction is supported in juvenile zebrafish *D. rerio*, in which growth depensation is prevented when food is superabundant [45], reducing competition for food.

Strategic growth is a growth pattern in which growth suppression (or growth restraint) is strongest when individuals are similar in size [21]. In Model 1, this is equivalent to *x* > 0. When decision making can evolve (Model 2), this pattern is evolutionarily stable whenever a stable solution can be found (e.g. electronic supplementary material, figures C.1–C.3). In Model 1, strategic growth was necessary but not sufficient for the emergence of stable but unequal size ratios. Such size ratios emerged when *x* > 0, opportunities for suppression were high, the effect of size on competition for food was relatively weak, and suppression had strong effects on growth; otherwise, size ratios converged to 1 or 0. The same conditions resulted in fewer size ratios close to 1 than expected by chance at the ESS in an evolutionary stochastic dynamic game (Model 2). Many systems in which strategic growth has been described or suggested (e.g. *P. xanthosomas*, [22]; *A. percula*, [2,5,41]; *Amphiprion ocellaris*, [6]; male *N. pulcher*, [4,21,43,52]; *C. bicolor* [42,53]) generally fit well with the models' predictions. All of these species live in long-term groups, so opportunities to interact are high. Further, spatial segregation, which may limit opportunities for dominants to interact with subordinates, is associated with lack of growth regulation and social instability (e.g. *Amphiprion frenatus* [54]; *C. bicolor* [42]). There is evidence that escalated conflict is more frequent among similarly sized individuals in systems with strategic growth [20,22,41,43] and that larger individuals are more likely to succeed in escalated contests (e.g. [20]). Although escalated conflict is not necessarily associated directly with growth suppression, and growth suppression could result in the absence of direct aggression, this suggests that the occurrence and outcome of conflict among group members in these systems depends on relative size.

Stable size differences exist only when individuals can suppress the growth of partners (e.g. *C, z, h* and *u* are greater than 0 in Model 1) and when investment in suppression is highest when size differences are small (*x* > 0 in Model 1). In several systems, larger individuals can influence the growth of smaller ones directly or indirectly [2,4,21,46]. Growth suppression in these systems does not require that dominants exclude subordinates from resources. In the goby *P. xanthosomas* subordinates refrain from eating when size differences are small [44]. Changes in growth rate are associated with changes in allocation of energy to reserves versus growth in *N. pulcher* [47]. Finally, social interactions, including submissive behaviour, are often energetically costly [55], and the frequency of submissive displays increases at smaller size differences [43], so changes in energy expenditure may contribute to growth suppression.

We assumed that growth regulation results from suppression and/or restraint. However, growth can be influenced by social context in other ways. In *N. pulcher*, newly ascended dominants grow rapidly [4,21,47]. This pattern is also observed in several highly social mammals [56–59] and in the cichlid, *Astatotilapia burtoni* [60] in which loss of rank is also associated with reduced growth. In anemonefish *A. percula* and meerkats *Suricata suricatta*, individuals increase their own growth rate when the growth rate of competitors for status is increased (‘competitive growth’, [61–63]). In many systems, growth rate increases after a period of growth restriction (‘compensatory growth’; [64–67]). The effects of status, competitive and compensatory growth were beyond the scope of our models. However, these mechanisms can influence the emergence and dynamics of size structure (e.g. [60]), and so including these in future examinations of socially regulated growth would provide a fuller picture of the role of social context on the emergence of size structure in groups.

In the dynamic game (Model 2), the frequency of eviction decreased when relative size had a strong effect on suppression (figure 3; electronic supplementary material, figure C.4), supporting the hypothesis that size-based growth suppression promotes group persistence [5]. The risks of eviction and the likelihood of finding new groupmates in the event of eviction influenced the evolution of suppression strategies (electronic supplementary material, figure C.3), size ratio distributions (electronic supplementary material, figure C.5) and the frequency of eviction (electronic supplementary material, figure C.5). Interestingly, a high probability of eviction in the model was associated with lower frequencies of eviction at equilibrium (electronic supplementary material, figure C.5b). As the probability of eviction increased, the range of sizes over which subordinates invested in suppression decreased (compare electronic supplementary material, figures C.3a and C.3g), while suppression by dominants remained largely unchanged. This resulted in decreased subordinate growth and less frequent tugs-of-war over growth. We found that increased availability of pathways to fitness outside of the pair increased risks of eviction. As likelihood of obtaining new partners after eviction increased, rates of eviction increased (electronic supplementary material, figures C.5d and C.5f). This result is consistent with other models and empirical studies that suggest that conflict is more frequent when there are outside options for dominants and/or subordinates [68,69].

While strategic growth as defined here is currently known in only a few systems, these models can provide insights into the influence of feedbacks between status and conflict on self-assembly of dominance hierarchies more generally. In our models, there is positive feedback between growth and growth suppression, such that success in conflict over growth results in lower susceptibility to future suppression and increased ability to control the growth of others. This feedback is similar to that inherent in winner and loser effects (e.g. [27]) in which the probabilities of winning (or losing) a fight increase after a previous win (or loss) [70] because of changes in access to resources [71] or changes in information about fighting ability and motivation to fight [9,70]. The effects of losing a fight are often stronger and persist longer than the effects of winning [70,72]. Winner–loser effects can promote the formation and stability of dominance-structured societies [27,73,74]. In our models, this positive feedback dampens as differences in size increase; this dampening is necessary for size ratios to stabilize at values other than 0 or 1. Winner and loser effects can decay over time in the absence of new information (e.g. [75]) and models of hierarchy formation incorporating dampening can yield stable dominance hierarchies [76]. Together, our models and those of winner–loser effect suggest that both positive feedback on differences between individuals in the form of effects on size (this model) or self-perception of fighting ability [70], and negative feedback in the form of decay in winner–loser effects [76] or suppression being restricted to individuals of similar size (this model) are sufficient to generate stable, asymmetrical hierarchies.

## Data accessibility

Matlab scripts are available from GitHub: https://github.com/IMHamilton1/GrowthFeedback.git.
